# Healthcare Applications and Services in Converged Networking Environments

**DOI:** 10.1155/2010/649597

**Published:** 2011-04-20

**Authors:** Tasos Dagiuklas, Maria G. Martini, Athanasios Demiris, George Anastassopoulos

**Affiliations:** ^1^Department of Telecommunication Systems and Networks, TEI of Messolonghi, 30300 Nafpaktos, Greece; ^2^CISM, Kingston University London, Penrhyn Road, Surrey KT12EE, UK; ^3^Micro2Gen Ltd., New Technology Park NCSR Demokritos, Agia Paraskevi, 15341 Athens, Greece; ^4^Medical Informatics Laboratory, Democritus University of Thrace, 68100 Alexandroupolis, Greece

Next generation wireless and wireline communication technologies for healthcare applications and services is a new and growing field to facilitate reliable, innovative, personalized, and high-quality healthcare applications and services. Advances in networking infrastructures (e.g., *HSxPA/WiMax/LTE*) are important in order to deliver healthcare applications and services regardless of the patient's physical location. Furthermore, continuous and personalized care solutions address the participation of patients in care and prevention processes and respond to the needs of elderly people by using sensors and advanced wireless monitoring tools. Finally, another area where the use of next generation communication technologies may foster rapid developments in healthcare is the support of the translation of cutting-edge research into clinical practice. 

The goal of this special issue is to report on cutting-edge research in converged communications and networking solutions for healthcare. Basic research papers were solicited, together with papers on systems integration or deployment, reflecting those aspects of research in communications for healthcare which are distinctly different from communications research in general.

The call for papers for this special issue attracted numerous responses from the research community, and submissions covered diverse areas of interest. After an extensive peer review process, nine papers have been selected for inclusion in this special issue, addressing requirements for e-Health services and applications, technologies and systems for telemedicine applications, and pilot implementations of telemedical systems. Several application areas have been addressed, ranging from cardiology to neurology and chronic pulmonary diseases, besides general purpose telemedicine platforms. In the following paragraphs, we briefly introduce the papers.

The article “*Using exploratory focus groups to inform the development of targeted COPD self-management education DVDs for rural patients*” by M. Stellefson et al. assessed the self-management learning needs, experiences, and perspectives of COPD patients treated at a Certified Federal Rural Health Clinic to inform the development of a COPD self-management DVD. Engaging rural communities in formal qualitative inquiries to describe COPD specific needs may lead to future use of educational technologies aimed at improving quality of life for rural populations.

The article *“e-SCP-ECG+ protocol: an expansion on SCP-ECG protocol for health telemonitoring—pilot implementation.*” by G. C. Mandellos et al., extends the use of SCP-ECG protocol in order to be included in health monitoring systems by introducing new sections into SCP-ECG structure for transferring medical and comprehensive demographic data. It also considers the pilot implementation of the new protocol as a software component in a Health telemonitoring system.

The article “*Implementation of compressed sensing in telecardiology sensor networks,*” by E. C. Pinheiro et al., presents an implementation of a compressed sensing paradigm in a wireless sensor network. The cardiovascular function monitoring was assessed and found to be very profitable. It was found that the approach between the two distant worlds of wireless sensor networks and compressed sensing is very interesting and may be further transposed to other areas of patient monitoring.

The article “*An empirical analysis of the current need for teleneuromedical care in German hospitals without neurology departments*” by G. W. Ickenstein et al. delivers an excellent example of the high value in the use of next generation communication technologies to address the lack of highly specialized expertise in rural areas. The article describes the need for setting up teleneurology infrastructure in order to support small hospitals in rural areas when treating patients with strokes and other acute conditions or syndromes urging for neurological or neurosurgical intervention. A large study in 119 acute hospitals without neurology departments in smaller towns in Germany (less than 100,000 inhabitants) clearly shows that the introduction of teleneurology is expected to increase quality of care and dicrease the need for unnecessary patient transports among other advantages.

The article “*Tile-Ippokratis: the experience of an ehealth platform for the provision of health care services in the island of Chios and Cyprus*” by H. Papadopoulos presents an integrated low-cost and ease-of-use ehealth platform for the provision of ehealth services for both patients with chronic diseases and patients who have been served by an intensive care unit. The platform based on the use of terrestrial and wireless communication infrastructures, supported teleconsultation and ehealth service.

The article “*Vascular neurology nurse practitioner provision of telemedicine consultations*” by B. M. Demaerschalk et al. investigates the possibility of delivering timely consultations in cases of acute stroke patients in emergency departments of rural areas within established hub and spoke networks. The study investigated the delivery of diagnosis and treatment both in terms of quality of diagnosis and treatment as well as overall duration of the procedure, clearly indicating that there is high value in telestroke consultations.

The article “*Telemedical support in patients with chronic heart failure: experience from different projects in Germany*” by A. Müller et al. addresses the very important application of telemedical monitoring in cases of chronic heart failure by reviewing seven related projects in Germany. The article emphasizes on the need for standardization, especially in order to be able to compare different telemonitoring solutions. Each of the projects addressed introduces a different approach in capturing and improving the situation of the affected patients, and the authors present these aspects for each one of the projects. 

The article “*Flexible macroblock ordering for context-aware ultrasound video transmission over mobile WiMAX*,” authored by M. G. Martini and C. T. E. R. Hewage, focuses on the exploitation of the characteristics of emerging network technologies and of recent coding standards for the joint design of the compression and transmission strategy in a telemedical system for real-time delivery of ultrasound video sequences. 

The article “*Analysis of QoS requirements for e-Health services and mapping to evolved packet system QoS classes*” by L. Skorin-Kapov and M. Matijasevic provides a framework for the provisioning of 3 GPP network architectures. It proposes a mapping of e-Health service types to standardized 3GPP QoS class identifiers in the evolved packet system (EPS).

In summary, this special issue includes papers that span diverse areas of interest including system development and specific e-Health applications, as well as technological foundations. 

The first two articles address requirements for eHealth services. Two articles address telemedicine technologies and systems. Finally, a group of articles describe telemedicine pilot implementations.

